# Selectively manipulating softness perception of materials through sound symbolism

**DOI:** 10.3389/fpsyg.2023.1323873

**Published:** 2024-01-08

**Authors:** Hamza Nalbantoğlu, Beyza Melis Hazır, Dicle N. Dövencioğlu

**Affiliations:** Department of Psychology, Middle East Technical University, Ankara, Türkiye

**Keywords:** sound symbolism, cross-modal perception, onomatopoeia, Turkish onomatopoeia, haptic (tactile) perception, material perception, softness perception

## Abstract

Cross-modal interactions between auditory and haptic perception manifest themselves in language, such as sound symbolic words: crunch, splash, and creak. Several studies have shown strong associations between sound symbolic words, shapes (e.g., Bouba/Kiki effect), and materials. Here, we identified these material associations in Turkish sound symbolic words and then tested for their effect on softness perception. First, we used a rating task in a semantic differentiation method to extract the perceived softness dimensions from words and materials. We then tested whether Turkish onomatopoeic words can be used to manipulate the perceived softness of everyday materials such as honey, silk, or sand across different dimensions of softness. In the first preliminary study, we used 40 material videos and 29 adjectives in a rating task with a semantic differentiation method to extract the main softness dimensions. A principal component analysis revealed seven softness components, including Deformability, Viscosity, Surface Softness, and Granularity, in line with the literature. The second preliminary study used 27 onomatopoeic words and 21 adjectives in the same rating task. Again, the findings aligned with the literature, revealing dimensions such as Viscosity, Granularity, and Surface Softness. However, no factors related to Deformability were found due to the absence of sound symbolic words in this category. Next, we paired the onomatopoeic words and material videos based on their associations with each softness dimension. We conducted a new rating task, synchronously presenting material videos and spoken onomatopoeic words. We hypothesized that congruent word-video pairs would produce significantly higher ratings for dimension-related adjectives, while incongruent word-video pairs would decrease these ratings, and the ratings of unrelated adjectives would remain the same. Our results revealed that onomatopoeic words selectively alter the perceived material qualities, providing evidence and insight into the cross-modality of perceived softness.

## 1 Introduction

Arbitrariness in language proposes a lack of any inherent connection between the sounds of words and their meanings (Imai et al., 2014); however, sound symbolism challenges this notion by asserting that there exists a non-arbitrary relationship between sounds and meaning for some words. A subset of these sound-symbolic words consists of onomatopoeia, words that sound just like the things they refer to. The onomatopoeic words are frequently used in sound-symbolism research since, contrary to the general belief, they are informative about the sounds they refer to and provide cross-modal information about the objects. For instance, the word “crispy” not only conveys the auditory characteristics of the sound produced by crispy objects but also suggests certain tactile and potentially visual properties associated with these objects.

One of the classical examples of these non-arbitrary sound symbolic relationships concerning shape perception is called the “Bouba-Kiki” effect (Köhler, 1929; Maurer et al., 2006; Ozturk et al., 2013; Peiffer-Smadja and Cohen, 2019). In the earlier findings, participants often associated angular objects with pseudowords “Kiki” and round objects with “Bouba” (Ramachandran and Hubbard, 2001). Since then, the effect has been demonstrated across different cultural backgrounds and language groups, implying the universality of sound symbolism (e.g., Bremner et al., 2013; Ćwiek et al., 2021).

In addition to these, Etzi et al. (2016) found that round-shaped sounds (as in “Bouba”) were more related to smoother textures, while sharp-transient sounds (as in “Kiki”) were more related to rougher textures. There is also evidence for strong associations between sounds and material perception in relatively recent research (Jousmäki and Hari, 1998; Watanabe et al., 2012; Sakamoto and Watanabe, 2018; Wong et al., 2022). For instance, Sakamoto and Watanabe (2018) found specific relationships between sounds and tactile ratings in the Japanese language, such as /p/, /b/, and /n/ consonants being more related to soft materials while /ts/ and /k/ being more related to hard materials. When they asked participants to generate sound-symbolic words in Japanese (including novel pseudowords) while touching a variety of materials, the results revealed that voiced consonants (e.g., /dz/ and /g/) were more likely to be associated with roughness and voiceless consonants (e.g., /ʦ/, and /s/) with smoothness. Further evidence supporting the crossmodal interactions between haptic and auditory signals comes from the Parchment-skin illusion (Jousmäki and Hari, 1998; Guest et al., 2002; Fujisaki et al., 2015), where an auditory signal’s frequency and sound levels alter tactile roughness perception. For instance, researchers simultaneously presented sounds of varying frequencies and levels while participants rubbed their hands; they found that higher frequencies and sound levels led to the perception of more paper-like and rougher skin (Guest et al., 2002).

In another study, Lo et al. (2017) conducted two experiments investigating the relationship of fricative-plosive consonants to spiky-curved shapes and to rough-smooth tactile surfaces. They found no relationship between the fricative and plosive sounds and spiky-curved shapes. However, their second experiment results revealed that participants were more likely to associate fricative-consonant speech sounds (i.e., /f/, /h/, /s/) with rough-textured materials and plosive-consonant speech sounds (i.e., /b/, /p/, /t/) with smooth-textured materials. The relationship between speech sounds and food texture was also investigated in the literature. For example, Hanada (2019) used Japanese onomatopoeic words to extract 15 perceptual dimensions of food texture, such as smoothness, adhesiveness, or wateriness. In a later study, Hanada (2023) investigated the fabrics’ haptic dimensions and feelings of luxuriousness and pleasantness using onomatopoeia in Japanese. In terms of perceived fabric luxuriousness and pleasantness, the likelihood of having the /s/, /h/, /m/, and /u/ sounds were found to increase, while the /k/, /g/, /z/, /p/, and /a/ sounds were found to decrease. They also reported that /k/, /g/, /z/, /p/, and /a/ sounds were more associated with the cheapness and unpleasantness of fabrics.

The studies above provide evidence of sound symbolism’s role in tactile perception. However, the characteristics of touch signals are not limited to the tactile information from two-dimensional textures: Haptic, visual, and auditory research shows that human softness perception has multiple dimensions from viscous, granular, deformable materials to materials with soft surfaces (Cavdan et al., 2019, 2021a; Dövencioğlu et al., 2022). These softness dimensions are also closely related to how humans interact with materials and surfaces in their environment using exploratory procedures, which refer to a set of hand movements used to explore various material properties (Lederman and Klatzky, 1987; Dövencioğlu et al., 2022). Some examples include using pressing for deformable materials, rotating for granular materials, and stroking for materials with soft surfaces.

The study of haptic perception using visual stimuli is shown to be a reliable method in different studies. In a review by Okamoto et al. (2013), the judgments of tactile texture perception are directly compared from tasks with visual stimuli and with haptic stimuli. Their findings suggested that these two modalities are perceived as similar in surface properties, with some unique dimensions to each stimuli type, such as glossiness only for the visual stimuli and temperature only for the haptic stimuli. Similarly, Baumgartner et al. (2013) conducted a study using the same 84 materials in visual and haptic stimuli and found that the perception of materials in visual space was comparable to the perception of materials in haptic space. In yet another study, Cavdan et al. (2021b) compared the softness judgments of participants for materials in haptic, static (image), and dynamic (video) stimulus conditions and found three common perceived softness dimensions: viscosity, granularity, and surface softness. Considering that haptic and visual modalities yield the same dimensions of perceived softness, material videos can be reliably used in the experiments instead of actual materials if the research question is relevant to these common dimensions of the two modalities.

These findings might suggest a connection between sound symbolism and the multidimensionality of perceived softness, especially concerning materials that go beyond textiles (e.g., Hanada, 2023). Studies that relate sound symbolism to material perception are mostly limited to the Japanese language, which has a diverse vocabulary of onomatopoeic words relating to tactile perception (Watanabe et al., 2012; Sakamoto and Watanabe, 2018; Wong et al., 2022). Turkish is also rich in the number of onomatopoeic words that are used colloquially. Many of these onomatopoeic words describe the softness-related properties of materials, such as “pıtır pıtır” (with a patter) for granular, “tiril tiril” (floaty) for silky, and “vıcık vıcık” (slushy) for gooey materials. Moreover, these words may even have subtle non-arbitrary variations, depending on the nature of the action or movement, such as “şarıl şarıl” for water that flows abundantly with splashing sounds, “şırıl şırıl” for water that flows pleasantly in a small amount but continuously, and “şorul şorul,” although a less common onomatopoeia, for water that flows abundantly and loudly (Zülfikar, 1995). Despite a significant number of examples, there is no research looking for systematic associations between softness perception and onomatopoeia in Turkish.

In the current study, we aim to explore the potential influence of Turkish onomatopoeic words on the perception of softness in everyday materials. To this end, we created congruent and incongruent pairs of videos of everyday materials and spoken Turkish onomatopoeic words for each softness dimension. We conducted two preliminary studies using the semantic differentiation method with (1) material videos and (2) onomatopoeic words. We extracted perceived softness factors from these studies and determined the congruency of onomatopoeic words and material videos based on their ratings for softness-related adjectives. Next, we used these pairs in Experiments 1 and 2. We hypothesized that pairing materials with onomatopoeic words would selectively alter their perceived material qualities, i.e., congruent pairs with high-rated onomatopoeic words would increase the mean ratings of adjectives related to the softness dimensions of materials, whereas incongruent pairs with low-rated onomatopoeic words would decrease the mean ratings.

## 2 Materials and methods

We conducted two preliminary studies (Supplementary Studies 1, 2) to select the material videos and onomatopoeic words that were used in Experiments 1 and 2. We used the semantic differentiation method in these preliminary studies with 29 and 21 softness-related adjectives, respectively. First, participants rated 40 material videos in an online study (Supplementary Study 1). Next, a different group of participants rated 27 onomatopoeic words in the lab (Supplementary Study 2). Principal Component Analyses revealed seven softness-related factors in the first and four softness-related factors in the second study. Stimulus selection based on these results is described below.

### 2.1 Ethics statement

The research was approved by the METU Human Subjects Ethics Committee and in accordance with the Declaration of Helsinki.

### 2.2 Experiment 1

Experiment 1 is conducted to determine the baseline judgments for the materials with a smaller set of adjectives. These judgments are then put to the test with congruent and incongruent onomatopoeic words in Experiment 2.

#### 2.2.1 Participants

We recruited 23 participants (*M* = 21.13, SD = 1.42, two males) for the experiment via the SONA research sign-up system. Participants gave informed consent before the experiment. After completion, they were compensated for their time with course credits. All participants had normal or corrected to normal vision. None of the participants reported hearing loss or any other auditory condition.

#### 2.2.2 Stimulus selection: common dimensions and adjectives

The selection of adjectives for this experiment was informed by the common factors extracted from Supplementary Study 1 to Supplementary Study 2. The four common factors were Viscosity, Surface Softness, Granularity, and Roughness (Table 1, first column). We selected 13 adjectives that loaded to the same factor in both studies (Table 1, second column). Specifically, we selected six adjectives for Viscosity (gelatinous, slimy, sticky, gooey, slippery, and moisturous), three adjectives for Surface Softness (silky, velvety, and hairy), three adjectives for Granularity (sandy, powdery, and granular), and one adjective for Roughness (roughened) dimensions.

**Table 1:** Adjectives, materials, and onomatopoeic words selected for each dimension.

Adjective dimensions	Adjectives	Ratings	Onomatopoeic words	Congruency	Materials
*Viscosity*	Gelatinous	High	Şap şap	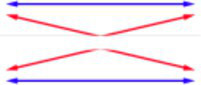	Shower gel	Low	Tak tak	Wool	Slimy	High	Şıp şıp	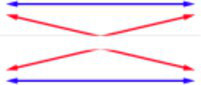	Honey	Low	Kütür kütür	Chickpeas	Sticky	High	Şapır şupur	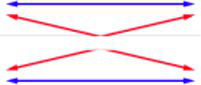	Slime	Low	Tangır tungur	Metal nuts	Gooey	High	Vıcık vıcık	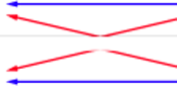	Hair conditioner	Low	Çıt çıt	Green lentils	Slippery	High	Şıpır şıpır	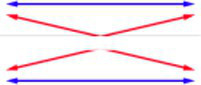	Olive oil	Low	Tıkır tıkır	Matchstick	Moisturous	High	Şarıl şarıl	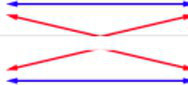	Hand cream	Low	Lime lime	Foam
*Surface Softness*	Silky	High	Tiril tiril		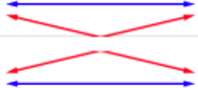	Silk	Low	Katır kutur	Scourer	Velvety	High		Mışıl mışıl	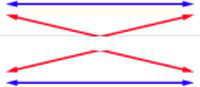	Velvet	Low	Kıtır kıtır	Stone	Hairy		High	Pofur pofur	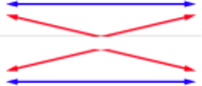	Fur	Low	Haşır huşur	Cardboard pieces																	
*Granularity*	Sandy	High	Hışır hışır	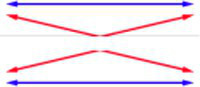		Sand	Low	Lıkır lıkır	Latex	Powdery	High	Püfür püfür	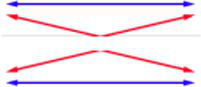		Flour	Low	Efil efil	Tulle	Granular	High	Pıtır pıtır	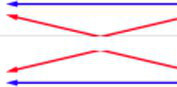		Poppy seeds	Low	Şırıl şırıl	Shaving cream
*Roughness*	Roughened	High	Kırt kırt		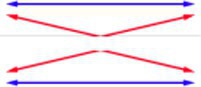	Sandpaper	Low	Gıcır gıcır	Balloon													

High- and low-rated onomatopoeic words and materials are listed for the corresponding adjectives. Blue arrows indicate congruent pairs (both high- or low-rated words and materials), whereas red arrows mark incongruent pairs (e.g., a low-rated word with a high-rated material).

From this adjective list, we continued to select the material videos. For each of the 13 adjectives, two material videos (one rated high and one rated low) from Study 1 were selected. A high rating was defined as a mean rating above 3.5 (out of 7) for a specific adjective. For instance, the shower gel video received a high rating for gelatinous with a mean score of 6.52 (SEM = 0.27). Conversely, a low rating was defined as a mean rating below 3.5. For example, the material video for wool received a low rating for gelatinous with a mean score of 1.35 (SEM = 0.12). The complete list of materials selected for the 13 adjectives can be found in Table 1. The resulting material videos also represented the same four dimensions with the adjectives. Overall, we selected 12 material videos for Viscosity, six material videos each for Surface Softness and Granularity, and two material videos for Roughness dimensions. The adjective scaly was not used for the Granularity dimension since all material ratings for scaly were lower than 3.5 (with a highest of 2.9), resulting in no high-rated materials.

#### 2.2.3 Procedure

**Figure 1:**
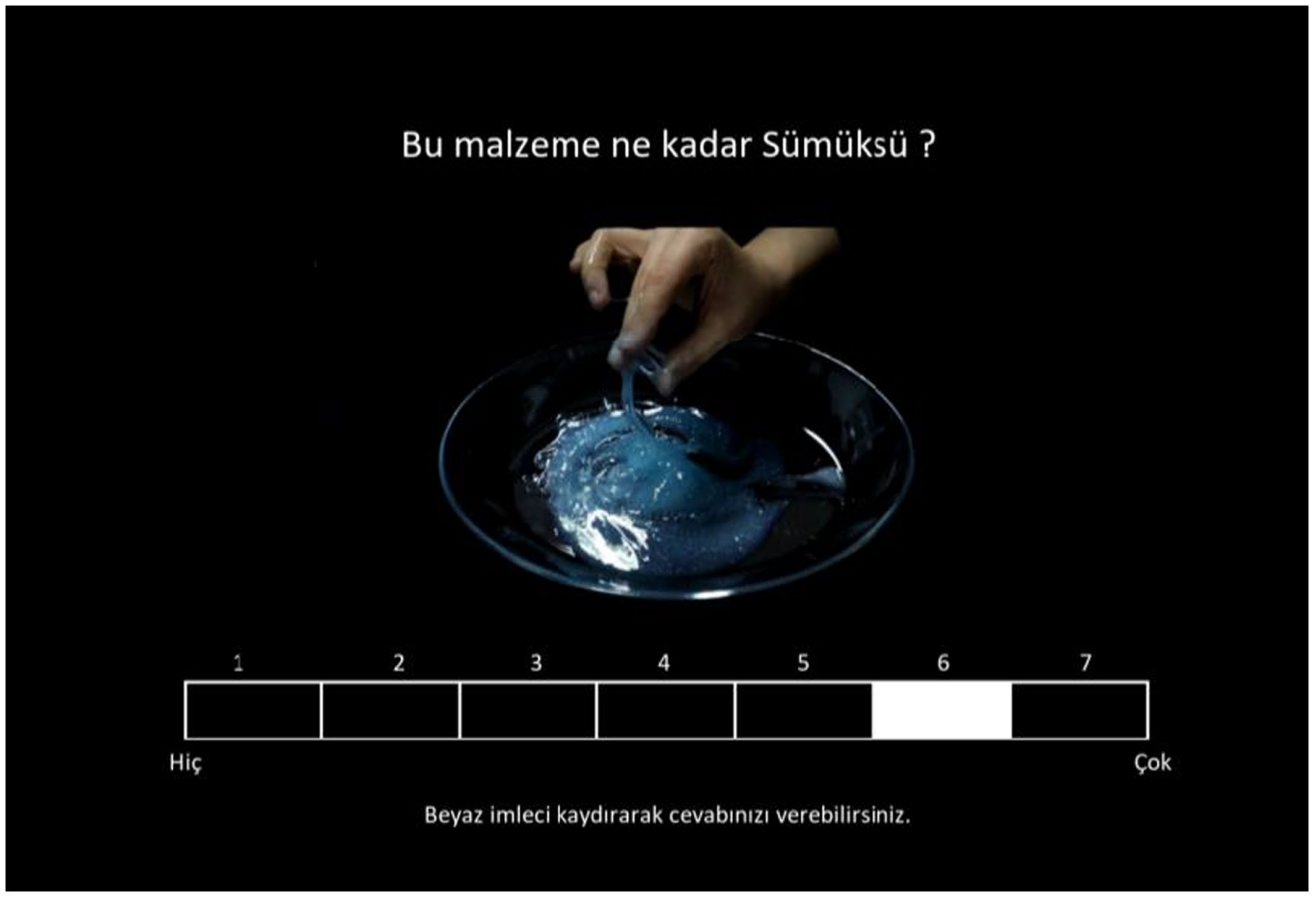
Screenshot from a sample trial in Experiment 1. While the material video of the “shower gel” is playing on the screen, participants are asked to rate the adjective “slimy (sümüksü)” from 1 to 7 based on the material.

The experiment was coded in MATLAB R2020b using Psychtoolbox and consisted of 26 material videos presented in a loop synchronously with the 13 adjectives one by one. One experimental block consisted of 338 trials for each participant. The experiment was conducted using an HP Pavilion TPC-F123-MT computer and HP 2011× 20-in LED Monitor. Participants were instructed to rate the adjectives they saw on the screen based on the material videos. A seven-point Likert scale was used for the ratings (1 = “*Not at all*,” 7 = “*Very*”). A sample trial from Experiment 1 is illustrated in Figure 1.

#### 2.2.4 Results

For each of the 13 adjectives, we calculated the mean ratings of the four material dimensions (Viscosity, Granularity, Surface Softness, and Roughness) and used them as baseline measurements for the mean ratings in Experiment 2 (Table 2; Figure 3, red lines). We averaged the ratings across within-dimension adjectives to come up with the adjective dimensions (Table 3, rows). Similarly, average ratings across the within-dimension materials gave us the material dimensions (Table 3, columns). All Adjective Dimensions, except for the Roughness Adjective, received the highest mean ratings from their own material dimensions (Table 3). Specifically, Viscosity Adjectives (*M* = 3.72, *SE* = 0.06) received the highest mean rating from the Viscosity Materials, Surface Softness Adjectives (*M* = 3.24, *SE* = 0.12) received the highest mean rating from Surface Softness Materials, and Granularity Adjectives (*M* = 3.83, *SE* = 0.14) had the highest mean rating for the Granularity Materials. However, Roughness Adjectives had the highest mean rating for the Surface Softness Materials (*M* = 4, *SE* = 0.17) and the second highest for Roughness Materials (*M* = 3.7, *SE* = 0.33).

**Table 2:** Mean ratings and standard errors of adjectives across material dimensions.

Adjectives	Material dimensions								Viscosity materials		Surface softness materials		Granularity materials		Roughness materials		Mean	SE	Mean	SE	Mean	SE	Mean	SE
Gelatinous	3.55	0.16	1.34	0.09	2.04	0.15	1.52	0.17
Slimy	3.59	0.16	1.25	0.06	2.01	0.15	1.24	0.11
Sticky	3.54	0.16	1.28	0.06	2.51	0.18	1.43	0.12
Gooey	3.80	0.16	1.12	0.03	2.39	0.17	1.17	0.10
Slippery	4.32	0.14	2.51	0.15	3.53	0.18	1.83	0.15
Moisturous	3.52	0.15	1.41	0.08	2.21	0.16	1.30	0.09
Silky	2.58	0.12	3.16	0.20	2.93	0.16	1.46	0.14
Velvety	2.38	0.10	3.46	0.21	2.85	0.16	1.85	0.21
Hairy	1.68	0.10	3.12	0.20	1.45	0.08	1.41	0.13
Sandy	2.17	0.11	1.70	0.12	3.98	0.23	1.35	0.15
Powdery	1.75	0.08	1.64	0.11	3.72	0.24	1.17	0.06
Granular	3.20	0.15	2.77	0.19	3.78	0.24	1.89	0.27
Roughened	2.62	0.12	4.00	0.17	3.30	0.18	3.70	0.33

**Table 3:** Mean ratings and standard errors of adjective dimensions across material dimensions.

Adjective dimensions	Material dimensions								Viscosity materials		Surface softness materials		Granularity materials		Roughness materials		Mean	SE	Mean	SE	Mean	SE	Mean	SE
Viscosity adjectives	3.72	0.06	1.48	0.04	2.45	0.07	1.42	0.05
Surface softness adjectives	2.21	0.06	3.24	0.12	2.41	0.09	1.57	0.09
Granularity adjectives	2.37	0.07	2.04	0.09	3.83	0.14	1.47	0.11
Roughness adjectives	2.62	0.12	4.00	0.17	3.30	0.18	3.70	0.33

When we look at the individual adjectives, we observe the same pattern of higher values for within-dimension ratings. All Viscosity Adjectives received the highest mean ratings from the Viscosity Materials (Table 2, leftmost column, first six adjectives). These adjectives were gelatinous (*M* = 3.55, *SE* = 0.16), slimy (*M* = 3.59, *SE* = 0.16), sticky (*M* = 3.54, *SE* = 0.16), gooey (*M* = 3.8, *SE* = 0.16), slippery (*M* = 4.32, *SE* = 0.14), and moisturous (*M* = 3.52, *SE* = 0.15). Similarly, silky (*M* = 3.16, *SE* = 0.2) and velvety (*M* = 3.46, *SE* = 0.21) had the highest mean ratings for the Surface Softness Materials, while sandy (*M* = 3.98, *SE* = 0.23) and powdery (*M* = 3.72, *SE* = 0.24) had the highest mean ratings for the Granularity Materials. Lastly, the adjective roughened, which is the only roughness adjective, received the highest mean rating (*M* = 4, *SE* = 0.17) from the Surface Softness Materials, but it received the second highest mean rating (*M* = 3.7, *SE* = 0.33) from the Roughness Materials.

Overall, the highest mean rating was observed for the adjective slippery (*M* = 4.32, *SE* = 0.14) for the Viscosity Materials, whereas the adjective gooey (*M* = 1.12, *SE* = 0.03) received the lowest mean rating with the Surface Softness Materials.

### 2.3 Experiment 2

#### 2.3.1 Participants

30 participants (*M* = 22.5, *SD* = 2.52, 11 Males) were recruited through the Middle East Technical University research sign-up system. Participants gave written informed consent before the experiment. After completion, they were compensated for their time with course credits. Only one participant was left-handed, and all participants had normal or corrected to normal vision. None of the participants reported hearing loss or any other auditory condition.

#### 2.3.2 Stimulus selection: onomatopoeic words

The adjectives and the material videos used in this experiment were the same as those used in Experiment 1. Additionally, we included 26 onomatopoeic words in this experiment. Similar to the selection process of the material videos in Experiment 1, two onomatopoeic words (one high-rated and one low-rated) were selected for each of the 13 adjectives based on the findings of Supplementary Study 2. A high rating was defined as a mean rating above 3.5 (out of 7) for a specific adjective. For instance, the onomatopoeic word “şap şap” received a high rating for gelatinous with a mean score of 4.7 (SEM = 0.36). Conversely, a low rating was defined as a mean rating below 3.5. For example, “tak tak” received a low rating for gelatinous with a mean score of 1.1 (SEM = 0.1). The complete list of onomatopoeic words selected for the 13 adjectives can be found in Table 1.

The material videos and onomatopoeic words were matched congruently (both are rated high, or both are rated low for a given adjective) or incongruently (one rated high when the other is rated low for a given adjective). We ended up with four material-onomatopoeic word pairs for each of the 13 adjectives (Table 1, blue lines for congruent, red lines for incongruent pairs).

#### 2.3.3 Procedure

We asked participants to rate material video-onomatopoeic word pairs for the same set of adjectives. For a total of 52 types of stimuli (13 adjectives × 2 previously received word ratings; high or low × 2 previously received material ratings; high or low), we collected 13 adjective ratings, resulting in 676 total trials. All participants took part in the experiment in a sound-isolated lab. The 5-s audio recordings of the onomatopoeic words were synchronously presented in a loop with the material videos using Sennheiser SK-507364 HD 206 headphones.

**Figure 2:**
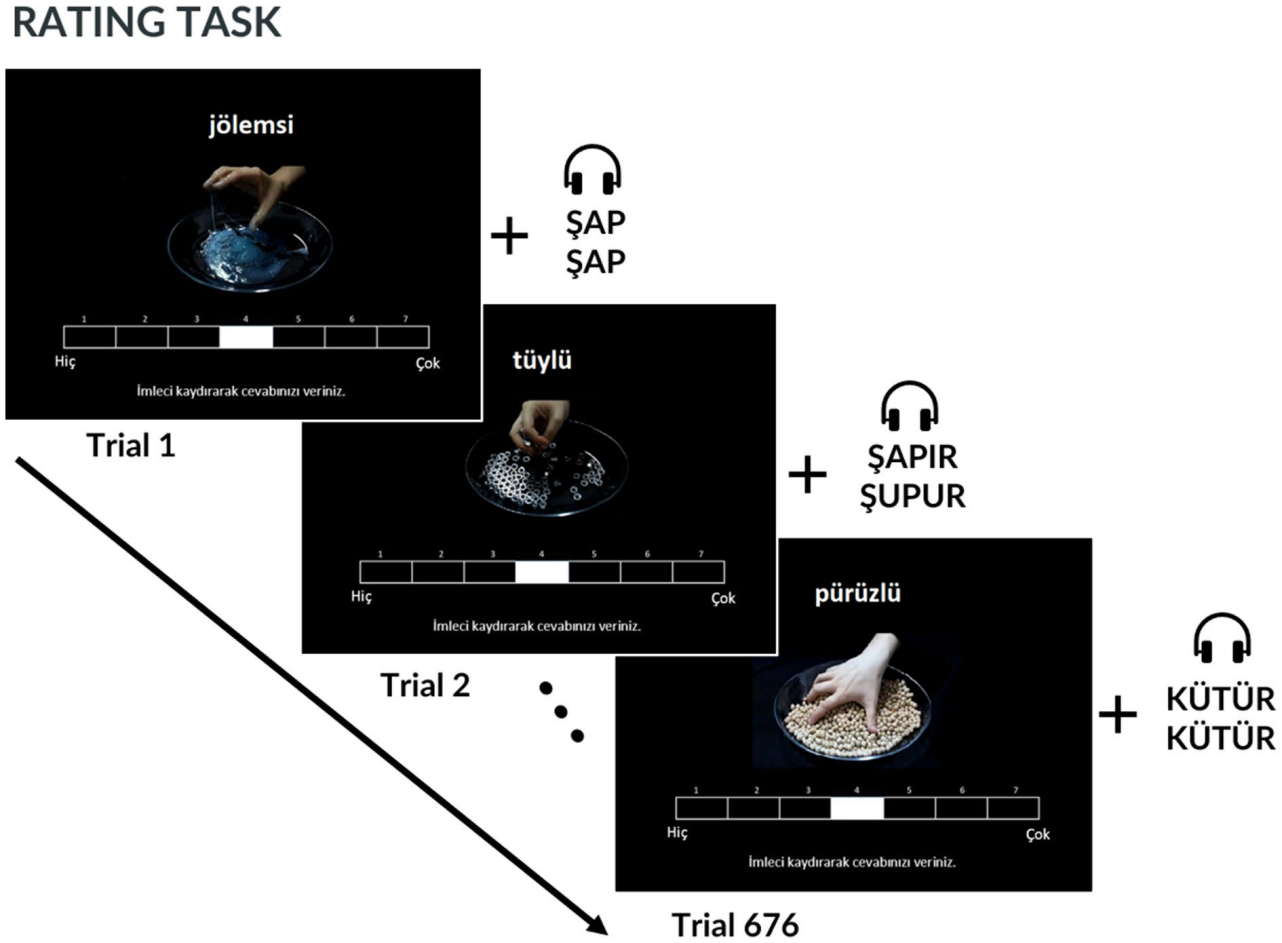
Procedure and timeline of Experiment 2. In each trial, participants watch a 5-s material video in a loop and also listen to the audio of the Turkish onomatopoeic words in a loop while they are rating the materials.

The ratings were collected on a seven-point Likert scale (1 = “Not at all,” 7 = “Very”) with a standard cable mouse. The experiment was coded in MATLAB R2020b using Psychtoolbox and conducted on an ASUS N550J Notebook. Participants were instructed to rate each pair of onomatopoeic words and material videos based on how well they thought the word-video pair matched the adjective displayed on the screen (Figure 2).

## 3 Results

All data analyses were performed using JASP (JASP Team, 2023). For analytical convenience, we grouped 26 individual material videos into one of the four softness dimensions (viscosity, surface softness, granularity, and roughness) and conducted the analyses at the dimension level rather than at the individual material level. A similar approach was adopted for the adjectives as well: viscosity (gelatinous, slimy, sticky, gooey, slippery, and moisturous), surface softness (silky, velvety, and hairy), granularity (sandy, powdery, and granular), and roughness (roughened) adjectives were grouped into their corresponding softness dimension. Analyses for individual adjectives were also conducted and presented in the Supplementary Analysis of Experiment 2.

We conducted four Repeated Measures ANOVAs for the adjective groups for each softness dimension. Since the groups violated the normality and sphericity assumptions, we report Greenhouse–Geisser corrected values. We also used Bonferroni correction for multiple comparisons and tested significance at α = 0.0018 level.

**Figure 3:**
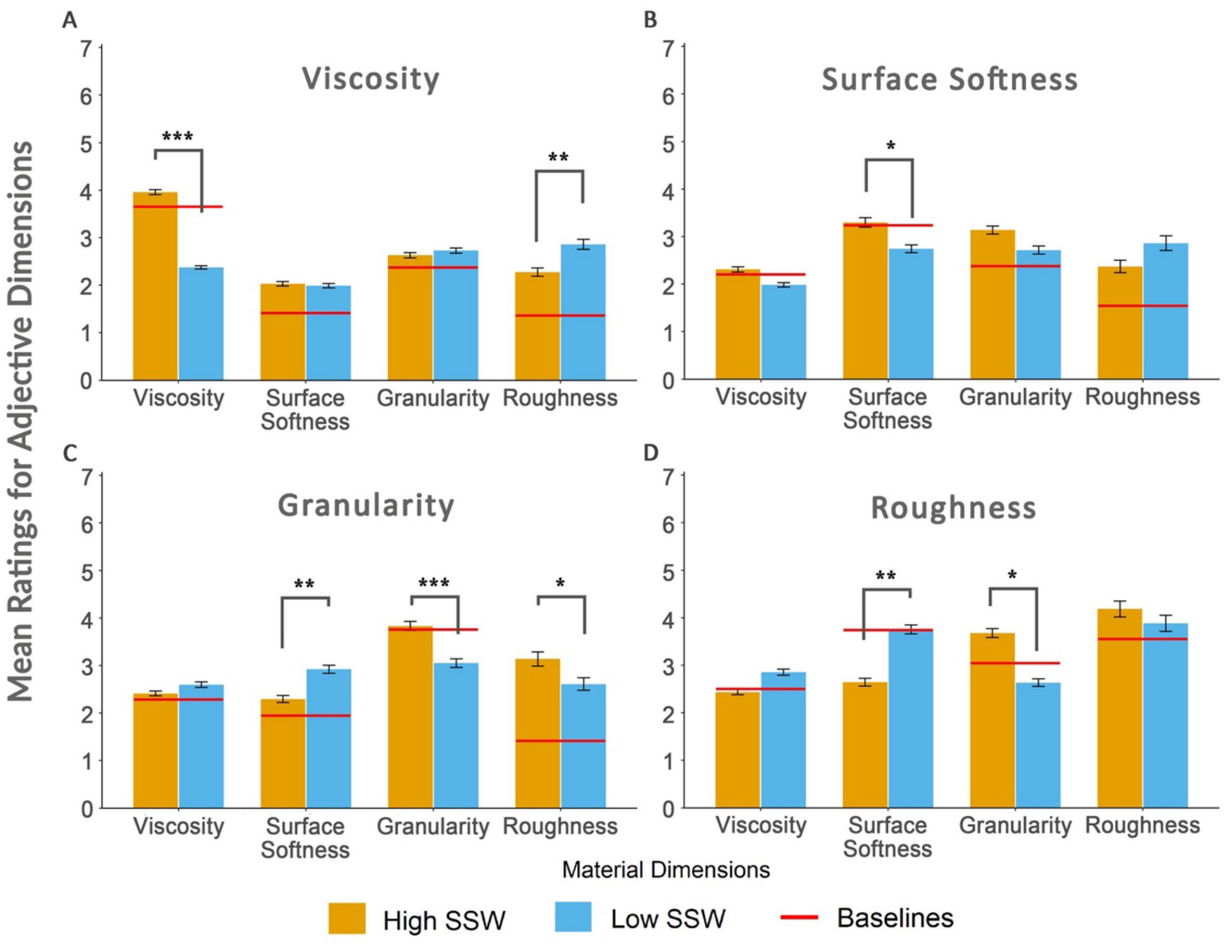
Mean ratings of Viscosity Adjectives **(A)**, Surface Softness Adjectives **(B)**, Granularity Adjectives **(C)**, and Roughness Adjectives **(D)** across different material dimensions. Yellow bars denote the conditions with high-rated onomatopoeic words while blue bars indicate the ones with low-rated onomatopoeic words. Red lines are baseline mean ratings for the corresponding material dimensions. ^*^p < 0.05; ^**^p < 0.01; and ^***^p < 0.001.

For the viscosity adjectives, main effects for the material dimension [*F*(1.86, 53.96) = 34.42, *p* < 0.001, η^2^_p_ = 0.54], and onomatopoeic word ratings [*F*(1, 29) = 25.31, *p* < 0.001, η^2^_p_ = 0.47] were significant. We also observed a significant interaction between the material dimensions and the onomatopoeic word ratings [*F*(1.96, 56.88) = 35.88, *p* < 0.001, η^2^_p_ = 0.55]. A *post hoc* analysis revealed that the mean ratings of viscosity adjectives were significantly lower for viscosity materials when they are paired with low-rated onomatopoeic words (*M* = 2.38, *SD* = 0.9) compared to when they are paired with high-rated onomatopoeic words (*M* = 3.96, *S* = 0.73; Figure 3A).

For instance, when the Viscosity Materials such as shower gel, wool, slime, and matchstick (Table 1) are presented with their respective low-rated onomatopoeic words (e.g., “tak tak, tangır tungur, or tıkır tıkır”), they are rated lower on the viscosity adjectives (e.g., gelatinous, slimy, gooey, and slippery) compared to when they are presented with their respective high-rated onomatopoeic words (e.g., “şap şap, şapır şupur, or şıpır şıpır”). On the other hand, the opposite effect was true for the roughness materials: We observed significantly higher mean ratings for the Viscosity Adjectives when they were paired with low-rated onomatopoeic words (*M* = 2.86, *SD* = 1.3) compared to when they were paired with high-rated onomatopoeic words (*M* = 2.28, *SD* = 1.11).

The surface softness adjectives also exhibited significant main effects for the material dimensions [*F*(2.61, 75.66) = 19.72, *p* < 0.001, η^2^_p_ = 0.41], and onomatopoeic word ratings [*F*(1, 29) = 6.61, *p* = 0.016, η^2^_p_ = 0.19]. In addition, a significant interaction effect was observed with these two factors [*F*(2.19, 63.61) = 9.01, *p* < 0.001, η^2^_p_ = 0.24; Figure 3B]. *Post hoc* analyses for surface softness adjectives revealed that mean ratings were significantly lower for the surface softness materials when they were paired with low-rated onomatopoeic words (*M* = 2.74, *SD* = 1) compared to when they were paired with high-rated onomatopoeic words (*M* = 3.3, *SD* = 1.1). However, this result was carried mainly by the adjective “silky” since it was the only one with a significant difference in mean ratings between high- and low-rated onomatopoeic words (see Supplementary Figure 5G).

In granularity adjectives, both main effects for the material dimensions [*F*(2.38, 69.02) = 22.02, *p* < 0.001, η^2^_p_ = 0.43] and onomatopoeic word ratings [*F*(1, 29) = 8.35, *p* = 0.007, η^2^_p_ = 0.22] showed significant differences. A significant interaction effect was also observed between material dimensions and word ratings [*F*(1.77, 51.25) = 14.82, *p* < 0.001, η^2^_p_ = 0.34; Figure 3C]. The *post hoc* analyses using Bonferroni correction revealed three groups with significantly different means for granularity adjectives. Firstly, similar to the Viscosity Adjectives and Surface Softness Adjectives, the mean ratings of Granularity Adjectives were significantly lower for granularity materials when they were paired with low-rated onomatopoeic words (*M* = 3.05, *SD* = 1.02) compared to when they are paired with high-rated onomatopoeic words (*M* = 3.83, *S* = 0.92). This effect is already evident in individual granularity adjectives (see Supplementary Figures 5J–L). Secondly, the mean ratings of Granularity Adjectives were significantly higher for the Surface Softness materials when paired with low-rated onomatopoeic words (*M* = 2.92, *SD* = 1.05) compared to when they were paired with high-rated onomatopoeic words (*M* = 2.29, *SD* = 1.01). This trend can be seen in the individual granularity adjectives in Supplementary Figures 5J–L, with only the adjective “granular” having a significant difference in the corresponding mean ratings. Lastly, the mean ratings of granularity adjectives were significantly lower for roughness materials paired with low-rated onomatopoeic words (*M* = 2.61, *SD* = 1.26) as opposed to when they are paired with high-rated onomatopoeic words (*M* = 3.14, *SD* = 1.36). Despite this difference, none of the individual granularity adjectives showed significant differences in ratings for roughness materials.

Finally, for the roughness adjective, significant main effects were observed for the material dimensions [*F*(2.8, 81.2) = 25.58, *p* < 0.001, η^2^_p_ = 0.47] and onomatopoeic word ratings [*F*(1, 29) = 0.2, *p* = 0.656, η^2^_p_ = 0]. Additionally, a significant interaction effect was observed between these two factors [*F*(1.64, 47.58) = 8.72, *p* = 0.001, η^2^_p_ = 0.23; Figure 3D]. The *post hoc* analysis results revealed that the mean ratings were significantly higher for surface softness materials when combined with low-rated onomatopoeic words (*M* = 3.76, *SD* = 1.47) compared to when they are combined with high-rated onomatopoeic words (*M* = 2.64, *S* = 1.24). In addition, mean ratings were significantly lower for granularity materials when they were presented with low-rated onomatopoeic words (*M* = 2.63, *SD* = 1.17) compared to when they were presented with high-rated onomatopoeic words (*M* = 3.68, *SD* = 1.14).

## 4 Discussion

The non-arbitrary nature of language is evidenced by associations of certain phonemes with different shapes and sizes across cultures. More recent findings even suggest nuances in phoneme-surface quality relationships in Japanese onomatopoeia (Sakamoto and Watanabe, 2018; Wong et al., 2022; Hanada, 2023). Here, we show for the first time that Turkish onomatopoeic words have unique associations with material softness qualities. Besides, the sound symbolism effect goes beyond surface material qualities to the perception of three-dimensional everyday materials. Finally, we demonstrate that spoken onomatopoeic words can be used to manipulate participants’ softness perception of everyday materials in a dimension-specific fashion. In two preliminary studies, we examined semantic spaces of Turkish onomatopoeic words and material videos with regard to the softness properties of materials. From these results, we created congruent and incongruent word-video pairs. Next, in two experiments, we used onomatopoeic words to selectively alter adjective ratings for materials. We observed increased ratings for the dimension-related adjectives of the congruent pairs with high-rated sound symbolic words and the opposite for incongruent pairs with low-rated sound symbolic words.

Cross-modal interactions between language and sensory processes provide some of the most striking examples of top-down influences on perception. One of the popular research topics on the subject, sound symbolism, has often focused on interactions between phonetic characteristics of words and shape perception (e.g., Bouba/Kiki effect). Only a few recent studies show a relationship between sound symbolic words and tactile material characteristics, and these are strictly conducted in Japanese (Watanabe et al., 2012; Lo et al., 2017). Similar to the haptic perception of materials, these studies also mostly focus on the surface properties of materials, with a couple of three-dimensional exceptions, such as compliant stimuli, i.e., springs (Sakamoto and Watanabe, 2017). Here, we add to surface softness properties and provide novel evidence for the overlap between multiple dimensions of perceived softness qualities of everyday materials and from Turkish sound symbolic words (SSWs). We extract four softness dimensions common to both materials and SSWs: Viscosity, surface softness, granularity, and roughness. This finding gives us the first insight into the multiple dimensions of softness via SSWs. One of the discrepancies with the literature is the lack of a deformability dimension for SSWs in our findings. Unlike material videos where we include deformable materials such as playdough, the word list in Supplementary Study 2 does not include any SSWs that correspond to the sound of a deforming material. To our knowledge, Turkish has no such examples, most likely because deforming materials do not make any sound (e.g., when compared to splashing water).

Next, we use these Turkish SSWs to manipulate the perceived softness qualities of materials along multiple softness dimensions. By pairing high- and low-rated onomatopoeic words with various materials, we observe changes in the ratings of adjectives related to the materials’ softness dimensions. Compared to baseline ratings (video-only stimuli), these dimension-specific changes are in the same direction as the ratings of the onomatopoeic words.

Our findings reveal fluctuations in dimension-specific adjective ratings predicted by the ratings for SSWs. Pairing viscosity materials with low-rated onomatopoeic words results in significantly lower ratings of viscosity-related adjectives (but not for adjectives in other dimensions) compared to when they were paired with high-rated words. Here, we also see a surprising effect, where the SSWs change the roughness materials’ ratings of slimy and slippery adjectives in an unexpected direction (Supplementary Figures 5B,E). Participants’ slimy and slippery ratings for “sandpaper” are higher when presented with a low-rated SSW for “roughened” (gıcır gıcır) compared to when presented with a high-rated SSW for “roughened” (kırt kırt). Except for “moisturous,” all other viscosity adjectives follow this trend as well, supporting the negative correlation between roughness and viscosity dimensions. An important observation for the viscosity adjectives dimension was that the effect of SSWs on the adjective ratings appeared to have a stronger diminishing impact than an enhancing one, which requires further research to understand the potential asymmetry of sound symbolism’s cross-modal effects.

For the surface softness dimension, SSW-material pairings only affected the ratings for surface softness materials significantly, as expected (Figure 3B). This result seems to be carried by the adjective silky: the ratings for velvety and hairy also differed in the expected direction, but these differences did not reach a significance level (Supplementary Figures 5G–I).

The SSWs altered all the granularity adjective ratings (sandy, powdery, and granular) in the expected direction for the granularity materials. The hairy ratings for granularity materials (Supplementary Figure 5I) were also affected similarly. Even though hairy is an adjective describing surface softness characteristics, it might indirectly be influenced by an SSW meaning, e.g., “püfür püfür” describing the gentle and refreshing wind that is blowing softly and coolly or “pıtır pıtır” (pitter patter) might have caused the granularity materials to be associated with the hairiness. Another unforeseen difference was the surface softness materials’ ratings for the granularity adjectives. Here, incongruent SSWs such as katır kutur or kıtır kıtır might mean a crunchy sound as in apple or a crispy sound as in cornflakes. Having this in mind, it is not surprising that for some participants, these SSWs resulted in higher semantic associations with being granular.

Finally, for the roughness adjective, both granularity and roughness materials showed effects in the expected direction, but the difference for roughness materials remained insignificant. Each dimension had different numbers of stimuli to be rated by the participants since our selection of adjectives, materials, and onomatopoeic words was based on the PCA results. This was especially true for roughness. As a result of the preliminary studies, we were able to choose only one adjective (roughened) with two corresponding material videos and two onomatopoeic words, compared to three to six adjectives in other dimensions. Thus, the insufficient number of ratings might have caused these results for the roughness adjective. This dimension is considered to be a control since it describes the surface qualities and not three-dimensional softness characteristics such as deformability (Dövencioğlu et al., 2022). So, our starting point to investigate sound symbolism for material qualities beyond surfaces might have led us to bring less focus to this dimension. Still, future research should aim to have a balanced number of stimuli across dimensions, if at all possible.

We also observed a significant difference in the mean ratings of the roughness adjective for the surface softness materials in the opposite direction to the roughness materials when paired with high and low-rated onomatopoeic words. This means that onomatopoeic words rated high in surface softness dimension decreased the roughness ratings of the surface softness materials -which is not surprising. This finding shows a clear contrast of the effects of sound symbolic words between the surface softness and roughness dimensions.

Another curious contrast we observed was in both the ratings for viscosity and surface softness adjectives. In both cases, we observed opposite effects for roughness materials, i.e., pairs with high-rated SSWs had lower rating values compared to the pairs with low-rated SSWs. This might be because a high-rated roughness material (sandpaper) paired with a low-rated SSW (gıcır gıcır meaning crisp., shiny, or brand new) might result in a higher rating for surface softness properties such as silky. It might also mean that the semantic association of the SSW and the material is conflicting, that the participants rated only one, either the SSW or the video. This distinction cannot be made conclusively from this study and needs further testing. Nevertheless, both types of contrasts suggest that a single SSW can sometimes provide information for more than one softness dimension, and sound symbolic effects on material perception are more complex than initially thought.

Lastly, our findings suggest an interesting parallel between the effects of sound symbolism on granularity and roughness adjectives when paired with their corresponding materials. This observation suggests a potential overlap between the two softness dimensions and brings up a new line of questioning for future research.

Japanese and Turkish sound symbolic words have discrepancies due to different vowel and consonant systems (Topbaş, 2007; Janhunen, 2023), or distinct combinations for word formation (Sakamoto and Watanabe, 2018; Kahraman and Akdağ, 2023) in two languages. For this reason, it is neither straightforward to compare our findings to previously reported literature nor is it in the scope of the current study. Nevertheless, an *ad hoc* comparison reveals common softness dimensions for viscosity (dry-wet component in Sakamoto and Watanabe, 2017, 2018), and roughness (rough-smooth component in Sakamoto and Watanabe, 2017, 2018). The reason for a lack of overlap might be due to the different lists of surfaces and materials in two studies. Unlike Sakamoto and Watanabe (2017), we avoid using adjectives such as comfortable, good, calm, etc. here to isolate sensory material characteristics.

One of the most prominent phonetic patterns in Turkish SSWs is represented by the strong effects in the viscosity dimension in our findings. Regardless of the vowels and suffixes, many Turkish SSWs involving the sound ş (/ʃ/), sometimes combined with r (/r/), relate to descriptions of water, where nuances describe the nature of the action or movement. One might speculate that the Turkish SSWs’ sh (/ʃ/) sound coincides with the /s/ sound which is found in Japanese SSWs (e.g., sara sara, syusa syusa) that have meanings correlated with slippery/moist dimensions. Despite the phonetic discrepancies between the two languages, small commonalities show promise for fruitful future research.

Overall, our results support the hypotheses that within-dimension pairs, matching materials with high-rated onomatopoeic words would enhance the mean ratings of adjectives related to the materials’ softness dimensions. In contrast, when materials are paired with low-rated onomatopoeic words, the mean ratings of the adjectives that describe the materials’ softness dimensions will decrease. Collectively, these findings deepen our understanding of the complex interplay between sound symbolism and material perception, particularly for perceived softness. They present promising pathways for further research while highlighting the unique role of onomatopoeic words in material perception. Our findings offer some potential applications in product marketing, virtual reality (VR), and augmented reality (AR) applications. For instance, the way sound-symbolic words influence the perceived softness of materials could provide new ways to enhance the consumer experience in online shopping as well as in advertising for boosting product descriptions with sound symbolism. In interfaces using VR or AR environments, sound symbolic words can be used to render a more realistic user experience with soft materials. This area of research may benefit from cross-cultural studies exploring both the phonetic features of onomatopoeic words and material perception. The universality of the Bouba/Kiki effect suggests that these phonetic effects might have commonalities in different cultures. The question of whether these findings are limited to Turkish speakers is a potential start for following research future investigations.

## Data availability statement

The datasets presented in this study can be found in online repositories. The names of the repository/repositories and accession number(s) can be found below: we have shared the data and the code and uploaded a sample video showing the experimental stimuli here: https://zenodo.org/records/10159924.

## Ethics statement

The studies involving humans were approved by METU Human Subjects Ethics Committee. The studies were conducted in accordance with the local legislation and institutional requirements. The participants provided their written informed consent to participate in this study.

## Author contributions

HN: Conceptualization, Data curation, Formal analysis, Investigation, Methodology, Project administration, Software, Visualization, Writing – original draft, Writing – review & editing, Validation. BH: Conceptualization, Data curation, Formal analysis, Investigation, Methodology, Project administration, Software, Validation, Visualization, Writing – original draft, Writing – review & editing. DD: Conceptualization, Formal analysis, Funding acquisition, Investigation, Methodology, Project administration, Resources, Supervision, Validation, Visualization, Writing – review & editing.
