# Supplementary material for: Selectively manipulating softness perception of materials through sound symbolism
Source: Front Psychol. 2024 Jan 8;14:1323873. doi: 10.3389/fpsyg.2023.1323873 (PMC10801190; doi:10.3389/fpsyg.2023.1323873)

***Supplementary Figure 1.* All Materials Used in Study 1.**


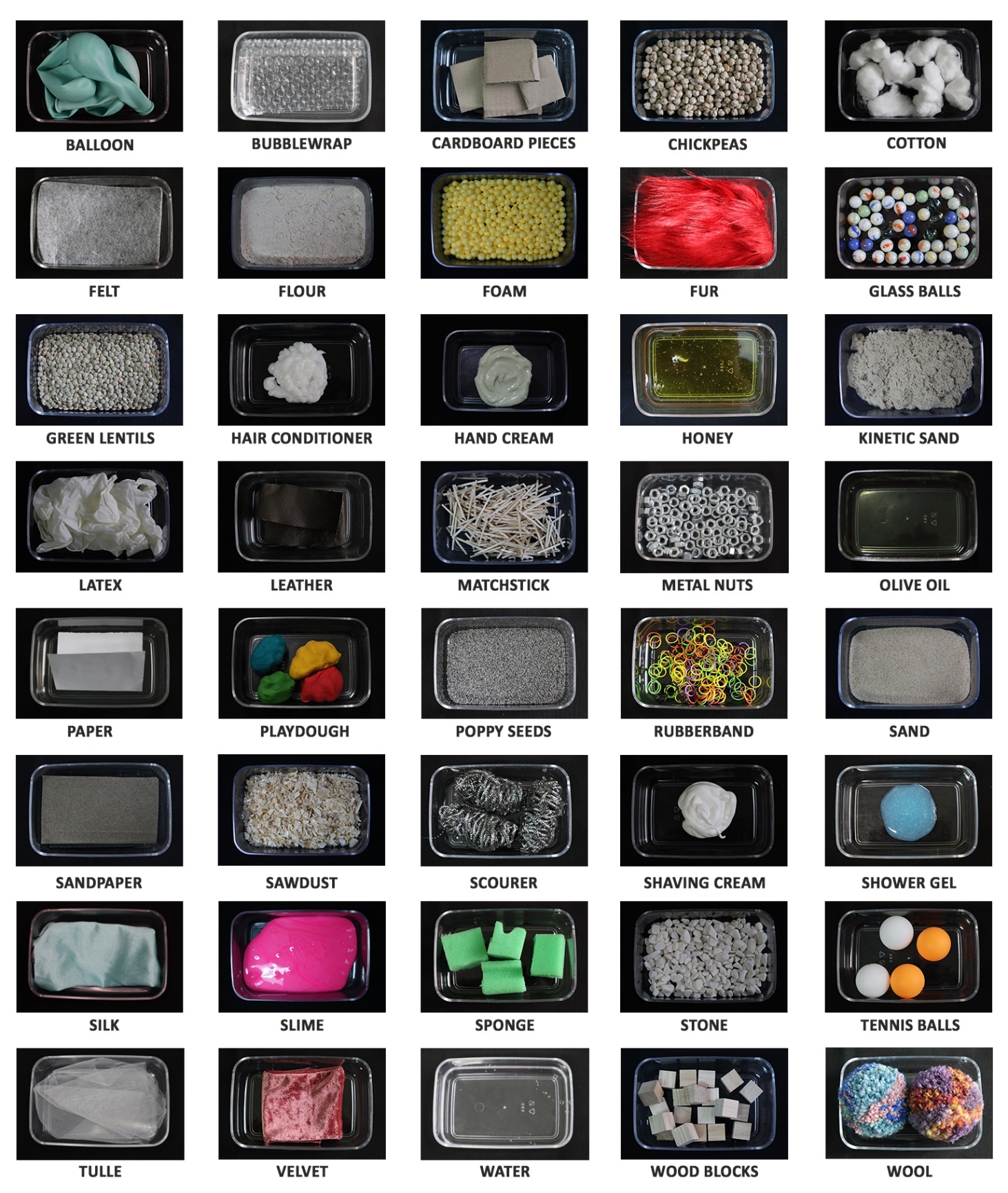


***Supplementary Table 1.* 29 Adjectives Used in Study 1 with Their English Translations.**

|  | **Adjective (TR)** | **Adjective (ENG)** |  | **Adjective (TR)** | **Adjective (ENG)** |
| --- | --- | --- | --- | --- | --- |
| 1 | *biçimlenebilir* | malleable | 16 | *odunsu* | woody |
| 2 | *dokulu* | textured | 17 | *parlak* | glossy |
| 3 | *esnek* | flexible | 18 | *pul pul* | scaly |
| 4 | *esnemez* | inflexible | 19 | *pürüzlü* | roughened |
| 5 | *güç uygulanabilir* | compliant | 20 | *sert* | hard/firm |
| 6 | *hamursu* | doughy | 21 | *sümüksü* | slimy |
| 7 | *hassas* | delicate | 22 | *süngerimsi* | spongy |
| 8 | *ipeksi* | silky | 23 | *tanecikli* | granular |
| 9 | *jölemsi* | gelatinous | 24 | *havadar* | airy |
| 10 | *kabarık* | fluffy | 25 | *toz gibi* | powdery |
| 11 | *kabuklu* | scabby | 26 | *tüylü* | hairy |
| 12 | *kadifemsi* | velvety | 27 | *cıvık* | gooey/sludgy |
| 13 | *kaygan* | slippery | 28 | *yapışkan* | sticky |
| 14 | *kum gibi* | sandy | 29 | *yumuşak* | soft |
| 15 | *nemli* | moisturous |  |  |  |

***Supplementary Figure 2.* Sample Single Trial from the Study 1.**

**
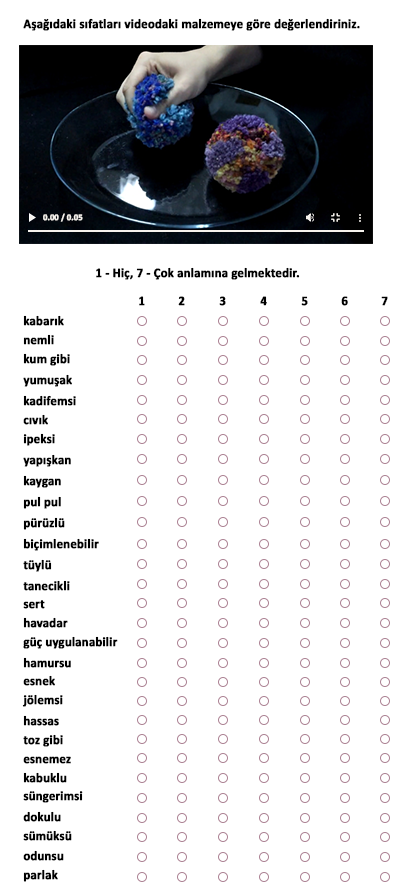
**

***Supplementary Table 2*. PCA Results for Study 1.**

|  | | **Components** | | | | | | | | | | | | | |  | |
| --- | --- | --- | --- | --- | --- | --- | --- | --- | --- | --- | --- | --- | --- | --- | --- | --- | --- |
|  | | **1** | | **2** | | **3** | | **4** | | **5** | | **6** | | **7** | | **Uniqueness** | |
| Gooey |  | 0.933 |  |  |  |  |  |  |  |  |  |  |  |  |  | 0.0417 |  |
| Gelatinous |  | 0.917 |  |  |  |  |  |  |  |  |  |  |  |  |  | 0.0688 |  |
| Slimy |  | 0.905 |  |  |  |  |  |  |  |  |  |  |  |  |  | 0.0814 |  |
| Sticky |  | 0.890 |  | 0.364 |  |  |  |  |  |  |  |  |  |  |  | 0.0540 |  |
| Moisturous |  | 0.870 |  |  |  |  |  |  |  |  |  |  |  |  |  | 0.1150 |  |
| Slippery |  | 0.800 |  |  |  |  |  |  |  |  |  | -0.378 |  | -0.341 |  | 0.0718 |  |
| Malleable |  |  |  | 0.926 |  |  |  |  |  |  |  |  |  |  |  | 0.0656 |  |
| Compliant |  |  |  | 0.896 |  |  |  |  |  |  |  |  |  |  |  | 0.0737 |  |
| Flexible |  |  |  | 0.826 |  |  |  |  |  |  |  |  |  |  |  | 0.1224 |  |
| Inflexible |  |  |  | -0.819 |  |  |  |  |  |  |  |  |  |  |  | 0.0899 |  |
| Doughy |  | 0.347 |  | 0.800 |  |  |  |  |  |  |  |  |  |  |  | 0.2243 |  |
| Delicate |  |  |  | 0.698 |  |  |  |  |  | 0.426 |  |  |  |  |  | 0.1368 |  |
| Soft |  | 0.427 |  | 0.680 |  |  |  | 0.379 |  | 0.348 |  |  |  |  |  | 0.0509 |  |
| Hard |  | -0.399 |  | -0.631 |  |  |  | -0.349 |  | -0.334 |  |  |  |  |  | 0.1172 |  |
| Sandy |  |  |  |  |  | 0.966 |  |  |  |  |  |  |  |  |  | 0.0587 |  |
| Powdery |  |  |  |  |  | 0.932 |  |  |  |  |  |  |  |  |  | 0.1050 |  |
| Granular |  |  |  |  |  | 0.890 |  |  |  |  |  |  |  |  |  | 0.0997 |  |
| Scaly |  |  |  |  |  | 0.726 |  |  |  |  |  | 0.414 |  |  |  | 0.1807 |  |
| Silky |  |  |  |  |  |  |  | 0.897 |  |  |  |  |  |  |  | 0.1223 |  |
| Velvety |  |  |  |  |  |  |  | 0.883 |  |  |  |  |  |  |  | 0.1683 |  |
| Hairy |  |  |  |  |  |  |  | 0.678 |  |  |  | 0.330 |  |  |  | 0.2257 |  |
| Airy |  |  |  |  |  |  |  |  |  | 0.835 |  |  |  |  |  | 0.1719 |  |
| Fluffy |  |  |  |  |  |  |  |  |  | 0.806 |  |  |  |  |  | 0.1451 |  |
| Spongy |  |  |  | 0.354 |  |  |  |  |  | 0.656 |  |  |  |  |  | 0.3081 |  |
| Roughened |  |  |  |  |  |  |  |  |  |  |  | 0.852 |  |  |  | 0.0989 |  |
| Textured |  | -0.307 |  |  |  |  |  |  |  |  |  | 0.851 |  |  |  | 0.1033 |  |
| Woody |  |  |  |  |  |  |  |  |  |  |  |  |  | 0.824 |  | 0.1523 |  |
| Scabby |  |  |  |  |  |  |  |  |  |  |  | 0.322 |  | 0.736 |  | 0.1669 |  |
| Glossy |  | 0.578 |  |  |  |  |  |  |  |  |  |  |  | -0.579 |  | 0.1803 |  |

*Note*. Varimax rotation was used.

***Supplementary Table 3.* Onomatopoeic Words Used in Study 2.**

|  | **Adjective (TR)** | **Explanation (ENG)** |
| --- | --- | --- |
| 1 | çıt çıt | Snap fastener, gripper |
| 2 | efil efil | Gently, intermittently, and slowly (blowing wind, snowing, hair waving) |
| 3 | gıcır gıcır | Crips, brand new |
| 4 | haşır huşur | Hard and dry things wring, wheezing, rumbling |
| 5 | hışır hışır | With a rustling sound |
| 6 | katır kutur | With a harsh and rough sound. |
| 7 | kırt kırt | Light, crisp sound, small or delicate breaking or cracking |
| 8 | kıtır kıtır | Crispy, brittle, crusty |
| 9 | kütür kütür | Crisp, fresh, with a crunching sound |
| 10 | lıkır lıkır | With a gurgling sound |
| 11 | lime lime | In small pieces, rags, and tatters |
| 12 | mırıl mırıl | Murmuring |
| 13 | mışıl mışıl | Sleeping peacefully and soundly, with a quiet and deep breath |
| 14 | pıtır pıtır | With a patter |
| 15 | pofur pofur | Soft, muffled, and continuous sound |
| 16 | püfür püfür | Light, gentle breeze or the sound of air softly moving or rustling |
| 17 | şap şap | Kissing with a screed sound, alum alum |
| 18 | şapır şupur | The sound of "smack-whisk" when kissing or eating |
| 19 | şarıl şarıl | Flowing splashingly, with a splashing sound |
| 20 | şıp şıp | Making a 'flashing' sound, plop |
| 21 | şıpır şıpır | Water droplets falling; a gentle, rhythmic dripping sound |
| 22 | şırıl şırıl | Continuous and loud flowing of water with a pleasant noise |
| 23 | tak tak | The sound that is made during hitting, impact, rat-tat |
| 24 | tangur tungur | Crash bang wallop, bone-shaking, clack |
| 25 | tıkır tıkır | At a rattling pace, tickety-boo |
| 26 | tiril tiril | Crisp and clean, gauzy, floaty |
| 27 | vıcık vıcık | Ropy, sludgy, gooey, slushy |

***Supplementary Figure 3*. Sample Trial from the Study 2.** The adjective “wet” is presented on the screen while the participant is hearing the onomatopoeic word from the headphone. A rating scale is presented to rate the adjectives using the cursor.


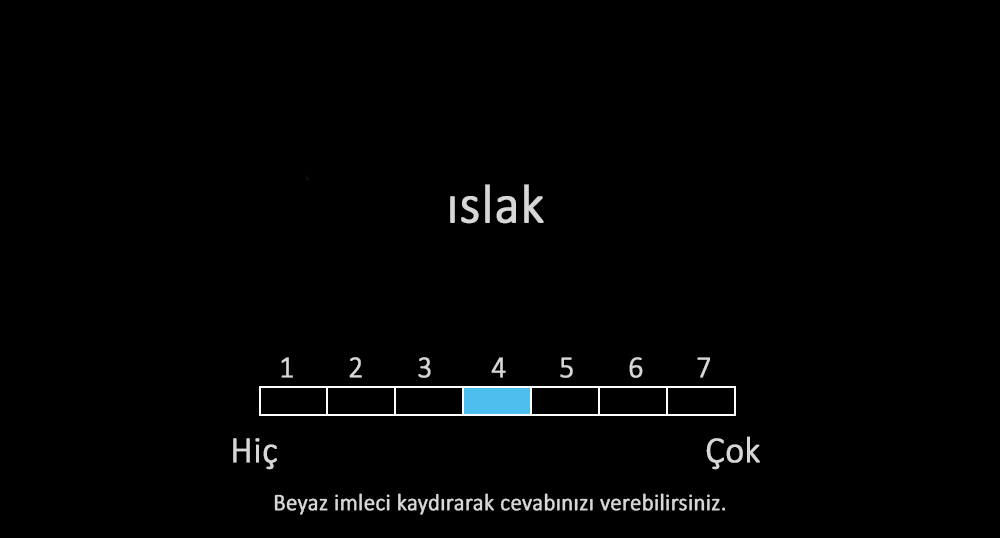


***Supplementary Table 4*. PCA Results for the Study 2.**

|  | | **Components** | | | | | | | |  | |
| --- | --- | --- | --- | --- | --- | --- | --- | --- | --- | --- | --- |
|  | | **1** | | **2** | | **3** | | **4** | | **Uniqueness** | |
| Sticky |  | 0.945 |  |  |  |  |  |  |  | 0.0502 |  |
| Slimy |  | 0.944 |  |  |  |  |  |  |  | 0.0410 |  |
| Gooey |  | 0.928 |  |  |  |  |  |  |  | 0.0142 |  |
| Gelatinous |  | 0.924 |  |  |  |  |  |  |  | 0.0818 |  |
| Moisturous |  | 0.873 |  |  |  | -0.324 |  |  |  | 0.0844 |  |
| Slippery |  | 0.837 |  |  |  |  |  | -0.421 |  | 0.0319 |  |
| Woody |  | -0.653 |  | -0.629 |  |  |  | 0.308 |  | 0.0465 |  |
| Roughened |  | -0.536 |  | -0.515 |  | 0.524 |  | 0.348 |  | 0.0528 |  |
| Velvety |  |  |  | 0.937 |  |  |  |  |  | 0.1011 |  |
| Hairy |  |  |  | 0.919 |  |  |  |  |  | 0.1009 |  |
| Silky |  |  |  | 0.912 |  |  |  |  |  | 0.1168 |  |
| Airy |  |  |  | 0.899 |  |  |  |  |  | 0.1552 |  |
| Fluffy |  |  |  | 0.824 |  |  |  |  |  | 0.1752 |  |
| Soft |  | 0.486 |  | 0.799 |  |  |  |  |  | 0.0482 |  |
| Hard |  | -0.604 |  | -0.688 |  |  |  |  |  | 0.0670 |  |
| Scabby |  | -0.609 |  | -0.619 |  | 0.343 |  |  |  | 0.0567 |  |
| Sandy |  | -0.327 |  |  |  | 0.915 |  |  |  | 0.0467 |  |
| Scaly |  | -0.328 |  | -0.347 |  | 0.824 |  |  |  | 0.0919 |  |
| Powdery |  |  |  | 0.393 |  | 0.802 |  |  |  | 0.0866 |  |
| Granular |  | -0.404 |  | -0.585 |  | 0.597 |  |  |  | 0.0772 |  |
| Glossy |  |  |  |  |  |  |  | -0.902 |  | 0.0766 |  |

*Note*. Varimax rotation was used.

***Supplementary Figure 4.* ANOVA and Post Hoc Results of Individual Adjectives.** (A) Red color gradient represents the F values of ANOVAs, with white being zero and increasing towards red. Asterisks indicate the p values (* p < .05 ** p < .01 *** p < .001). (B) Red color gradient represents the mean differences in the post hoc tests, with red being a positive difference and decreasing towards -0.9 in grey. Asterisks indicate the p values (* p < .05 ** p < .01 *** p < .001).


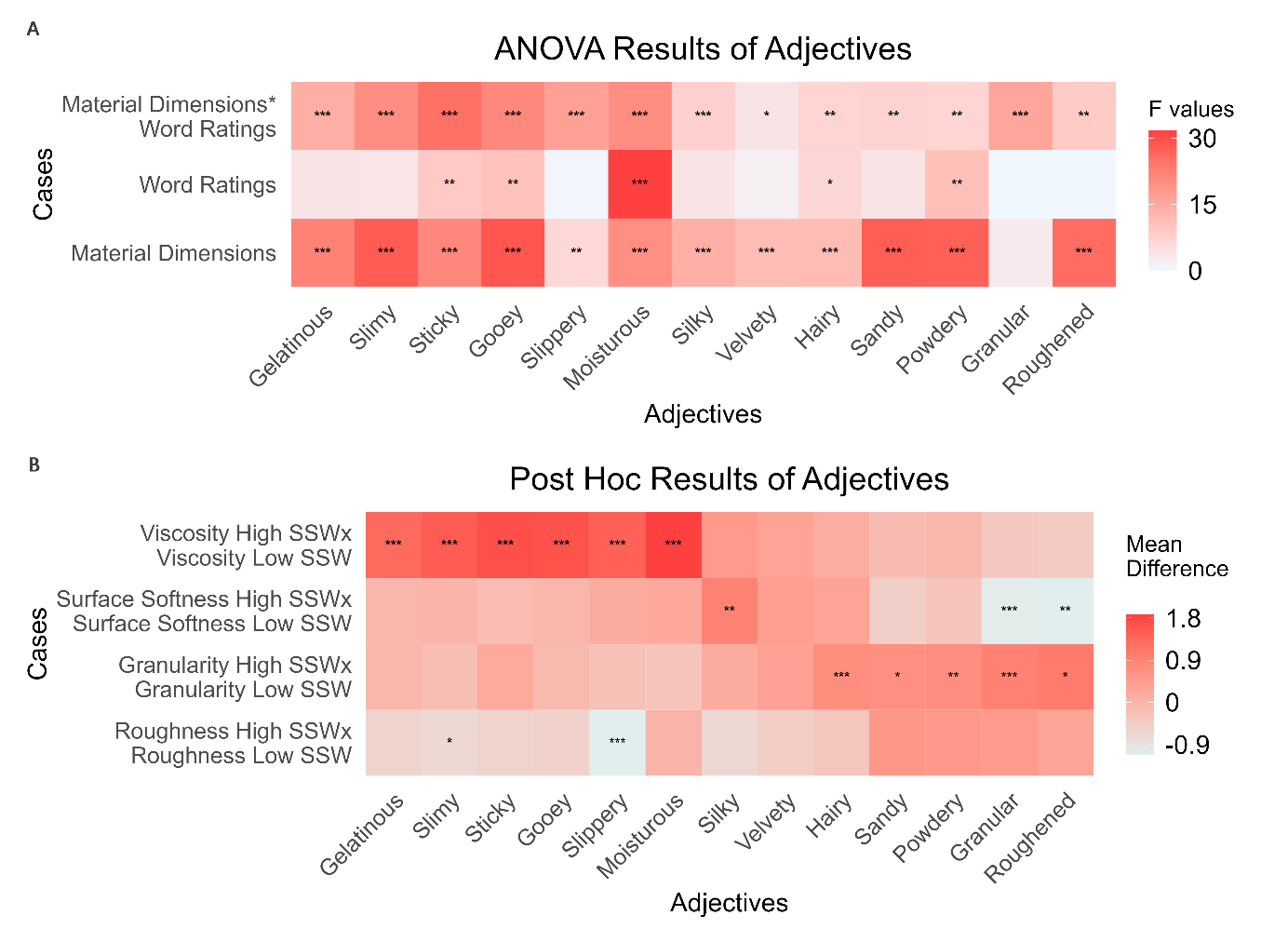


***Supplementary Figure 5.* Ratings of Individual Adjectives Across Different Cases.** Yellow bars denote the conditions with high-rated onomatopoeic words while blue bars indicate the ones with low-rated onomatopoeic words. Red lines are baseline mean ratings for the corresponding material dimensions. * p < .05 ** p < .01 *** p < .001


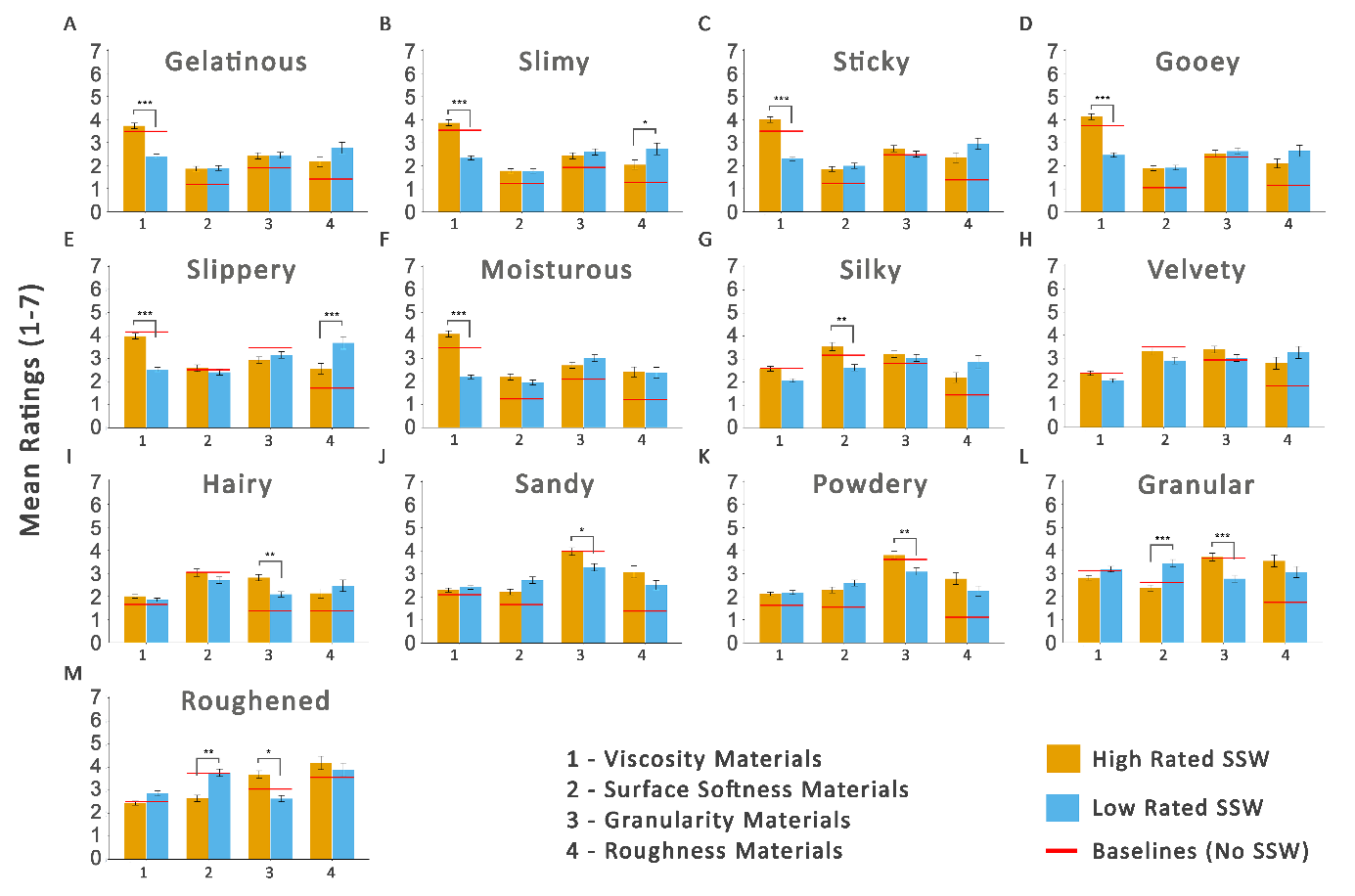

Supplement: Supplementary file 1 [file Data_Sheet_1.docx]
