# Supplementary material for: Selectively manipulating softness perception of materials through sound symbolism
Source: Front Psychol. 2024 Jan 8;14:1323873. doi: 10.3389/fpsyg.2023.1323873 (PMC10801190; doi:10.3389/fpsyg.2023.1323873)
Supplement: Supplementary file 2 [file Data_Sheet_2.docx]

**1 Supplementary Study 1**

**1.1 Participants**

15 university students (*M* = 23.8, *SD* = 4.5, 2 Males) volunteered to participate in the online study (Qualtrics) through the Middle East Technical University research sign-up system. All participants were naive to the purposes of the study. Only one participant was left-handed. Participants gave written informed consent before the study and were debriefed and given course credit after they had completed the study.

**1.2 Stimuli**

**1.2.1 Material Videos**

We used material videos as stimuli as they are reported to be assessed as comparable to haptic stimulus judgments (Baumgartner et al., 2013; Cavdan et al., 2021; Okamoto et al., 2013). Thirty-two soft and 8 hard materials (as control) were selected (see Supplementary Figure 1 for the complete list of materials). Videos showed materials in their glass containers and experimenters' hand movements against a black background. Videos were recorded without sound using Canon EOS M50 with a tripod placed 50 cm away from the materials. Natural light and automatic ISO settings were used. The spatial resolution was set to 1920x1080 pixels Full HD, and videos were recorded at 50 frames per second. Next, 5-second video clips were cut for the best representative exploratory procedures that present the most salient feature of a material, such as pressing for sponge pieces (deformable), rubbing for velvet (textile), stirring for hand cream (viscous, Dovencioğlu et al., 2022).

**1.2.2 Adjectives**

The list of 31 adjectives used here is taken from a previous study that was initially adapted to Turkish by Dovencioğlu et al. (2019; 2022) from Guest et al. (2011). This list consisted of non-sensual adjectives that relate to material characteristics of soft and rough materials. We only removed 2 adjectives from the list, “meaty” and “leathery." The former was not included since it was not closely related to our material list, and the latter was not included since the Turkish translation of the adjective was not a commonly used word in the daily language. The complete list of the resulting 29 adjectives and their English translations can be found in Supplementary Table 1.

**1.3 Procedure**

The experiment was conducted online on Qualtrics due to the global COVID-19 pandemic restrictions. Participants were instructed to play the 5-second video of the material and then to rate the materials shown in the videos based on the adjectives. Overall, they completed 40 blocks with 29 adjectives each for one material video. They were required to complete all ratings before proceeding to the subsequent trial. Adjectives were rated on a 7-point Likert scale (1 = "*Not at all*”, 7 = “*Very appropriate*”). Supplementary Figure 2 depicts a sample trial from the study.

**1.4 Results**

Before the analysis, we ran Barlett’s test of sphericity (χ2(406) = 1546, *p* < .001) and Kaiser-Mayer-Olkin (KMO) test (score = .584) to make sure that the data can be used in a Principal Component Analysis. The PCA was conducted to see the adjective dimensions using JAMOVI (The jamovi project, 2019). Using the Kaiser normalization and varimax rotation, 7 principal components were extracted, explaining 87.58% of the total variance in the data. The component loadings are shown in Supplementary Table 2.

The first component, Viscosity, accounted for 21.03% of the variance and was characterized by the adjectives *gooey, gelatinous, slimy, sticky, moisturous,* *slippery,* and *glossy.* The second component, Deformability, accounted for 20.32% of the variance and comprised the adjectives *malleable, compliant, flexible, inflexible (-), doughy, delicate, soft,* and *hard (-)*. The third component, Granularity, explained 12.43% of the variance and included the adjectives *sandy, powdery, granular*, and *scaly*. The fourth component, Surface Softness, accounted for 9.17% of the variance and included the adjectives *silky*, *velvety*, and *hairy*. The fifth component, Fluffiness, explained 8.93% of the variance and consisted of the adjectives *airy, fluffy,* and *spongy*. Roughness, the sixth component, explained 8.4% of the variance and included the adjectives *roughened* and *textured*. Lastly, the seventh component, Scabbiness, accounted for 7.3% of the variance and was characterized by the adjectives *woody* and *scabby.*

**2 Supplementary Study 2**

**2.1 Participants**

30 university students (*M* = 22.4, *SD* = 2.4, 7 males) volunteered through the Middle East Technical University research sign-up system. Three participants were left-handed, and all participants had normal or corrected to normal vision. None of the participants reported a hearing loss or any other auditory condition. All participants were native Turkish speakers and naive to the purposes of the study. An informed consent was provided before the study, and participants received course credits after completion.

**2.2 Stimuli**

**2.2.1 Spoken Onomatopoeic Words**

First, 51 material-related onomatopoeic words were chosen from Turkish linguistics literature (Zulfikar, 1995; Ozkan, 2010). After that, we checked the meanings of these words from the Turkish Language Society, TDK (Türk Dil Kurumu) dictionary. We also checked the frequencies of the words using TS Corpus, the largest Turkish corpora (Sezer, 2017; Sezer & Sezer, 2013). Those with a frequency of 0 were eliminated since they might be unfamiliar to the participants. We completed another online rating study to determine the familiarity of the words and further eliminated the words with an average familiarity rating below 3 (out of 7). In the last step, we removed the words that are not related to our material list (e.g., “vız vız” referring to the sounds of the bees), resulting in a list of 27 onomatopoeic words (see Supplementary Table 3 for the complete list of onomatopoeic words).

One of the experimenters (BMH, a native Turkish speaker) recorded the spoken onomatopoeic words using a RODE noise-canceling microphone. The recordings took place in a soundproof room and were edited using Audacity software (Audacity Team, 2021). The audio files started with a 1-second silent onset before the onomatopoeic words (~2 seconds), followed by a silent offset time to complete the recordings to a duration of 5 seconds. The audio recordings were normalized for volume level consistency in the final step.

**2.2.2 Adjectives**

The adjective list used in Study 1 is shortened to 21 in Study 2 to optimize experiment runtime. Except for the deformability related ones (2, 3, 4, 5, 6, 7, and 22nd adjectives) that are excluded from the adjective list of this study, the adjectives used in Study 2 are listed in the Supplementary Table 1.

**2.3 Procedure**

The study was coded in MATLAB R2020b (The MathWorks Inc., 2020) using Psychtoolbox-3 and included 27 spoken onomatopoeic words along with 21 adjectives. Participants rated every onomatopoeic word for 21 adjectives, resulting in a total of 567 randomized trials for a single experimental session. The experiment was conducted using an HP ENVY dv6 Notebook in a sound-isolated room. The 5-second audio recordings of the onomatopoeic words were presented to the participants synchronously with their written forms on the screen in a loop, using Sennheiser SK-507364 HD 206 headphones. Ratings were collected with a standard cable mouse after the first 3 seconds of the recordings to ensure that the participants listened to the recordings. A 7-point Likert scale was used for the ratings (1 = "*Not at all”*, 7 = “Very”). All participants were instructed to listen to the audio first and then rate the onomatopoeic word for the adjective they saw written on the screen. A sample trial is depicted in Supplementary Figure 3.

**2.4 Results**

A Principal Component Analysis (PCA) was conducted to see the resulting adjective dimensions using JAMOVI (The jamovi project, 2019). Barlett’s test of sphericity was significant (χ2(210) = 995, *p* < .001), and the Kaiser-Mayer-Olkin (KMO) test resulted in a score of .535, indicating that the data is suitable for a PCA. Using the Kaiser normalization and varimax rotation, 4 principal components were extracted. The resulting component loadings are presented in Supplementary Table 4.

The first component, Viscosity, accounted for 35.02% of the variance, and it was characterized by the adjectives *sticky, slimy, gooey, gelatinous, moisturous,* and *slippery.* The second component, Surface Softness, accounted for 32.91% of the variance and comprised the adjectives *velvety, hairy, silky, airy, fluffy, soft, hard (-), and scabby (-)*. The third component, Granularity, explained 16.85% of the variance and included the adjectives *sandy, scaly, powdery*, and *granular*. The fourth component, Roughness, explained 7.85% of the variance and included the adjectives *woody, roughened,* and *glossy (-)*.

**3 Supplementary Analysis: Results for Individual Adjectives**

First, we analyzed the effects of high and low-rated onomatopoeic words on individual adjectives across 4 different material dimensions. The results of Shapiro-Wilk tests and Mauchly's sphericity tests showed that assumptions of normality and sphericity had been violated in all groups for 13 adjectives. We conducted 13 Repeated Measures ANOVAs using Greenhouse–Geisser adjustment and Bonferroni correction for multiple comparisons (resulting in α = .05/13 = .003). All ANOVA results except for the adjective “granular” showed a significant main effect of material dimensions (see Supplementary Figure 4A for the ANOVA results of adjectives). Only the adjectives “sticky, gooey, moisturous, hairy, and powdery” showed significant main effects of onomatopoeic word ratings. All ANOVA results showed significant interactions between the material dimensions and ratings of the onomatopoeic words. To determine which specific material dimensions and onomatopoeic word ratings caused these significant interaction effects, we conducted 4 post hoc tests for each of the 13 adjectives (see Supplementary Figure 4B).

There were significant differences between the mean ratings of all viscosity adjectives (gelatinous, slimy, sticky, gooey, slippery, and moisturous) for the viscosity materials that were matched with high or low-rated onomatopoeic words. For example, the mean ratings of the adjective "gelatinous" were lower for the viscosity materials, including "shower gel, green lentils, and hand cream" (see Table 1 for the complete list) when they were presented with their respective low-rated onomatopoeic words such as "tak tak, çıt çıt, or lime lime” as opposed to when they were presented with the high rated onomatopoeic words such as “şap şap, vıcık vıcık or şarıl şarıl” (see Supplementary Figure 5A). The adjectives “slippery” and “slimy” also had significant differences in their mean ratings for the roughness materials (Supplementary Figure 5B and 5E). More specifically, the mean ratings of "slimy" and "slippery" were significantly higher for the roughness materials (sandpaper and balloon) when they were presented with the low-rated onomatopoeic word "gıcır gıcır” compared to their mean ratings when they are presented with the high rated onomatopoeic word “kırt kırt." For the surface softness adjectives (silky, velvety, and hairy), only in "silky," there was a significant difference between the mean ratings for the surface softness materials when they were paired with high or low-rated onomatopoeic words (Supplementary Figure 5G). For the adjective "hairy," these differences in the mean ratings were observed for the granularity materials when they were paired with high or low-rated onomatopoeic words (Supplementary Figure 5I).

There were also significant differences between the mean ratings of all granularity adjectives (sandy, powdery, and granular) for the granularity materials that were matched with high or low-rated onomatopoeic words. For example, the mean ratings of the adjective "powdery" were lower for the granularity materials (i.e., sand, poppy seeds, latex) (Table 1) when paired with a low-rated onomatopoeic word such as “lıkır lıkır, efil efil, or şırıl şırıl” compared to when paired with a high-rated onomatopoeic word such as “hışır hışır, püfür püfür, or pıtır pıtır” (Supplementary Figure 5K). There was also a significant difference between the mean ratings of "granular" for the surface softness materials when paired with a high or low-rated onomatopoeic word (Supplementary Figure 5L).

Finally, neither low- nor high-rated onomatopoeic words affected the ratings of roughness materials for the adjective “roughened." However, there were significant differences in the ratings of surface softness and granularity materials when paired with high or low-rated onomatopoeic words (Supplementary Figure 5M). For example, the Surface Softness materials (i.e., silk, stone, or velvet) received significantly lower mean ratings for the adjective "roughened" when matched with low-rated onomatopoeic words compared to high-rated onomatopoeic words. All remaining results for the individual adjectives are illustrated in Supplementary Figure 4 and Figure 5.
